# Understanding hand hygiene behaviour in the intensive care unit to inform interventions: an interview study

**DOI:** 10.1186/s12913-020-05215-4

**Published:** 2020-04-25

**Authors:** Kathryn Lambe, Sinéad Lydon, Caoimhe Madden, Jenny McSharry, Rebecca Marshall, Ruth Boylan, Aoife Hehir, Molly Byrne, Omar Tujjar, Paul O’Connor

**Affiliations:** 1 Discipline of General Practice, School of Medicine, National University of Ireland Galway, Co. Galway, Ireland; 2 Irish Centre for Applied Patient Safety and Simulation, School of Medicine, National University of Ireland Galway, Co. Galway, Ireland; 3 School of Medicine, National University of Ireland Galway, Co. Galway, Ireland; 4 School of Psychology, National University of Ireland Galway, Co. Galway, Ireland; 5 Department of Anaesthesia & Intensive Care, University Hospital Galway, Co. Galway, Ireland; 6 The College of Anaesthesiologists of Ireland, Dublin, Ireland; 7 Department of Anaesthetics, Sligo University Hospital, Sligo, Ireland

**Keywords:** Hand hygiene, Infection control, Compliance, Intensive care, Interview, Behaviour

## Abstract

**Background:**

Improving hand hygiene (HH) compliance is one of the most important, but elusive, goals of infection control. The purpose of this study was to use the capability (C), opportunity (O), motivation (M), and behaviour (B; COM-B) model and the theoretical domains framework (TDF) to gain an understanding of the barriers and enablers of HH behaviours in an intensive care unit (ICU) in order to identify specific interventions to improve HH compliance.

**Methods:**

A semi-structured interview schedule was developed based upon the COM-B model. This schedule was used to interview a total of 26 ICU staff: 12 ICU nurses, 11 anaesthetic specialist registrars, and three anaesthetic senior house officers.

**Results:**

Participants were confident in their capabilities to carry out appropriate HH behaviours. The vast majority of participants reported having the necessary knowledge and skills, and believed they were capable of carrying out appropriate HH behaviours. Social influence was regarded as being important in encouraging HH compliance by the interviewees- particularly by nurses. The participants were motivated to carry out HH behaviours, and it was recognised that HH was an important part of their job and is important in preventing infection. It is recommended that staff are provided with targeted HH training, in which individuals receive direct and individualised feedback on actual performance and are provided guidance on how to address deficiencies in HH compliance at the bedside at the time at which the HH behaviour is performed. Modelling of appropriate HH behaviours by senior leaders is also suggested, particularly by senior doctors. Finally, appropriate levels of staffing are a factor that must be considered if HH compliance is to be improved.

**Conclusions:**

This study has demonstrated that short interviews with ICU staff, founded on appropriate behavioural change frameworks, can provide an understanding of HH behaviour. This understanding can then be applied to design interventions appropriately tailored to the needs of a specific unit, which will have an increased likelihood of improving HH compliance.

## Background

Improving hand hygiene (HH) compliance is one of the most important, yet elusive, goals of infection control [1]. Although the HH procedure itself is simple, the behaviours related to consistent engagement with HH are complex and not readily understood, explained, or changed [2, 3]. A commonly used approach to improve compliance is to use a bundled HH intervention whereby several strategies are used together [2, 4]. However, this method may not constitute the most effective use of limited resources and there is a need to consider all interventional strategies employed, their necessity and sufficiency [2, 4, 5]. It has been suggested that the development and/or the selection of interventions to implement changes in healthcare practice is often conducted on the basis of intuition [6–8], and, correspondingly, very few studies of interventions to improve HH compliance are grounded in behaviour change theory [9]. Therefore, to best identify how to improve HH compliance, we first require an understanding of the determinants of current and desired behaviours informed by a theory of behavioural change.

There are many different theories of behaviour change, often with similar and overlapping constructs [10]. The diversity and number of these theories have been identified as possible reasons why theoretical approaches are not used in the design of interventions [11]. In response to these issues, the Theoretical Domains Framework (TDF) was developed in order to provide a structure to support the application of theoretical approaches to interventions with the goal of fostering behavioural change [12, 13]. The framework supports an evidence-based approach to the identification of interventions that are most likely to bring about changes in behaviour. The TDF consists of 14 domains. It was designed using a systematic consensus approach to simplify and amalgamate the numerous behavioural change theories relevant to behavioural change [13]. The TDF has been used as a framework to study barriers to HH in the past [3, 14–17].

A related model is the COM-B model, in which three factors interact to generate behaviour: the individual’s capability (C), opportunity (O), and motivation (M) to engage in a given behaviour (B). The COM-B model is a compilation of 19 different behavioural change frameworks and complements the TDF as a tool for understanding behaviour and supporting development of appropriate interventions [11]. The benefit of the COM-B model over other, more established theories is that it is designed to be simple yet comprehensive and describes the minimum number of factors needed to account for behaviour change. As can be seen from Table 1, each of the COM-B domains maps onto several unique TDF domains.

**Table 1:** Theoretical domains framework and COM-B domains (adapted from Michie et al., 2014 [10])

COM-B	TDF Domain	Definition
**Capability**	Knowledge	An awareness of the existence of something.
	Skills	An ability or proficiency acquired through practice.
	Beliefs about capabilities	Acceptance of the truth, reality, or validity about outcomes of a behaviour in a given situation.
	Behavioural regulation	Anything aimed at managing or changing objectively observed or measured actions.
	Memory, attention, & decision processes	The ability to retain information, focus selectively on aspects of the environment and choose between two or more alternatives.
**Opportunity**	Social influences	Those interpersonal processes that can cause individuals to change their thoughts, feelings, or behaviours.
	Environmental context and resources	Any circumstance of a person’s situation or environment that discourages or encourages the development of skills and abilities, independence, social competence and adaptive behaviour.
**Motivation**	Social/professional role and identity	A coherent set of behaviours and displayed personal qualities of an individual in a social or work setting.
	Optimism	The confidence that things will happen for the best or that desired goals will be attained.
	Intentions	A conscious decision to perform a behaviour or a resolve to act in a certain way.
	Goals	Mental representations of outcomes or end states that an individual wants to achieve.
	Beliefs about consequences	Acceptance of the truth, reality or validity about an ability, talent or facility that a person can put to constructive use.
	Reinforcement	Increasing the probability of a response by arranging a dependent relationship, or contingency, between the response and a given stimulus.
	Emotion	A complex reaction pattern, involving experiential, behavioural, and physiological elements, by which the individual attempts to deal with a personally significant matter or event.

The purpose of the study described in this paper was to use the COM-B model and the TDF as an analytical framework to gain an understanding of the barriers and enablers of HH behaviours in an intensive care unit (ICU) setting through semi-structured interviews with ICU healthcare providers. Qualitative methods support a ‘deeper’ understanding of healthcare workers’ perceptions of barriers and enablers of hand hygiene compliance than is possible using quantitative methodologies. A qualitative paradigm using semi-structured interviews allows participants to describe their experiences and issues in their own language, and gives them the flexibility to describe the unique insights of their professional group, which may not be obvious to a researcher outside that group [18].

Based upon the analysis of these interviews, suggestions for the types of interventions that may be effective in improving the HH compliance of healthcare providers in ICU will be briefly identified. In this way, our intention is to present a novel, user-friendly method, grounded in behavioural change theory, that will allow infection control and quality improvement teams to explore, diagnose and understand the barriers and enablers to HH compliance in their own units and hospitals, and thereby prescribe appropriate interventions to address their local challenges in a targeted way.

## Method

### Research design

This qualitative study used semi-structured interviews with ICU doctors and nurses. The study has been reported in accordance with the Standards for Reporting Qualitative Research (SRQR) [19].

### Setting

The participants were healthcare providers from two ICUs in hospitals in the Republic of Ireland. The ICUs had 25 and 5 beds respectively.

### Ethical considerations

Ethical approval was obtained from the ethics boards of both participating hospitals. All participants provided written informed consent prior to the interview.

### Participants

A total of 26 participants (12 ICU nurses, 11 anaesthetic specialist registrars, and three anaesthetic senior house officers) completed interviews as part of this study. There were no individuals approached during recruitment who declined to participate. Sixteen participants were female, 10 were male.

### Interview schedule

The semi-structured interview schedule (see Additional file 1) was developed based upon the COM-B model. Accordingly, questions were developed relating to participants’ capability, opportunity, and motivation to engage in HH behaviour. The capability questions asked participants about the training they had received and about their confidence in their knowledge of HH protocols. The opportunity questions were designed to assess whether sufficient resources were available to make HH compliance possible (e.g. time, facilities), and whether the social environment supported HH behaviour (e.g. other healthcare providers engage in HH). The motivation questions were concerned with assessing the respondents’ belief in the utility of HH, and whether they, and others in the unit, strive for high levels of appropriate HH behaviour.

The COM-B model was used to develop the interview guide, rather than the TDF, which was later used as a coding framework for the analysis. Following discussion with ICU staff as part of the development of the interview protocol, it was believed that the TDF was too constrictive to use to develop the interview guide and may lead participants to provide specific provide opinions about the topic that fit into the framework. That is, asking questions about barriers and enablers more broadly, under the COM-B headings, would allow comments about environmental resources, social support, and other factors to arise naturally, rather than being “prompted” by more narrow questions about individual domains of the TDF. This had the added advantage of keeping the interview schedule relatively short, as it did not have to cover all the domains of the TDF individually. The more comprehensive and granular TDF was later used as a coding framework for analysis. The interview protocol was piloted with two nurses and a doctor and found to be acceptable.

### Procedure

The interviews were carried out by three of the authors (one psychologist and two medical doctors) between February 2017 and November 2017. Participants were recruited using purposive sampling and snowball techniques. The psychologist did not know the participants that she interviewed. However, the two doctors did know the participants that they interviewed. Interviews were conducted at a place and time convenient to the interviewee. Interviews were conducted in person, audio recorded and transcribed. Audio recordings of the interviews were destroyed after transcription. Interviews were conducted on-site. The median duration of the interviews was 4 min 41 s. A stopping criterion of two interviews was used for doctors and nurses. The stopping criterion refers to the number of interviews to be conducted with in each sample group without new themes emerging. The sample frame included nurses and doctors who worked in ICU.

### Analysis

To ensure rigour and trustworthiness of the data, content analysis was performed by two researchers, both psychologists who had not been involved in recruitment for, or conducting of, the interviews. No software was used to support the analysis. The researchers annotated printed copies of each interview as they worked through the content analysis process. A deductive content analysis approach was used to analyse the interview data, in which the analysis was planned and operationalised based on existing knowledge with the goal of testing a given theory [20]. The TDF was used as the initial framework for classifying the interview data. The reason for using the TDF, as opposed to the COM-B domains, was due to the extra granularity in coding provided by the TDF domains. After having read and coded the interviews independently, the two coders compared their codes and resolved disagreements through discussion to achieve consensus. Following completion of the analysis, exemplar quotes for each of the domains were chosen by consensus between the two researchers. The number of interviewees that made comments related to each of the 14 TDF domains was also recorded.

## Results

The number and percentage of participants who made comments relating to each of the 14 TDF domains is provided in Table 2. It was found that the TDF was appropriate for analysing the data and all of the comments made by the participants could be classified using the TDF. A discussion of each of the TDF domains with exemplar comments made by the participants are provided below under the associated dimensions of the capability, opportunity and motivation dimensions of the COM-B model.

**Table 2:** Number and percentage of comments made by participants (*n* = 26) corresponding with each TDF domain

TDF Domain	No of participants who mentioned domains (%) (n = 26)
**Capability dimension**	
- Knowledge	22 (84.6%)
- Skills	25 (96.2%)
- Beliefs about capabilities	16 (61.5%)
- Behavioural regulation	3 (11.5%)
- Memory, attention, and decision processes	11 (42.3%)
**Opportunity dimension**	
- Social influences	21 (80.8%)
- Environmental context and resources	26 (100%)
**Motivation dimension**	
- Social/professional role & identity	14 (53.9%)
- Optimism	0
- Intentions	0
- Goals	4 (15.4%)
- Beliefs about consequences	19 (73.1%)
- Reinforcement	12 (46.2%)
- Emotion	7 (26.9%)

### Capability dimension

#### Knowledge

The majority of participants (*n* = 22, 84.6%) confirmed knowledge of the reasons for, and importance of, HH compliance. For example, one participant noted that “*hand hygiene protocols are important in health care industry just for prevention of cross-infection*” (P3, Doctor).

#### Skill

All but one (*n* = 25, 96.2%) all of the interviewees described training in hand hygiene and were satisfied with the HH compliance training they had received. Several (*n* = 15, 57.7%) also described the necessity for ongoing top-up training. It was expressed by one of the doctors that “*in this hospital there was about two structured training sessions with one infection control (person) and the second one with nursing staff* “ (P20, Doctor).

#### Beliefs about capabilities

Some of the doctors and nurses interviewed expressed different levels of confidence in their ability to carry out appropriate HH behaviours. Confidence may be higher among physicians, who were more likely to express confidence in their own HH knowledge and skills as compared to nurses. To illustrate, a doctor stated that *“I’m confident in my knowledge of my hand hygiene protocols and I don’t feel I need a lot more hand hygiene protocols to be taught to me”* (P4, Doctor), whereas a nurse said, *“you can never have enough. It’s always good to be reminded and refreshed”* (P15, Nurse).

#### Behavioural regulation

This category was used infrequently (see Table 2). Only three participants (11.5%) reported checking their own behaviour or consciously reminding themselves to perform HH. To illustrate, one of the nurses said, “*once you have this patient stable, then you have to look back and say what did we do well here? ... So you’re always reviewing what you’ve done in the context of could I have caused an infection to this patient*?” (P25, Nurse).

#### Memory, attention, and decision processes

Nurses in particular were more likely to describe HH as a habitual behaviour (*n* = 5 nurses (41/7%), *n* = 1 doctor (7.1%)). This point is illustrated by the following quote: *“you just do it unknown to yourself”* (P12, Nurse). However, 11 (42.3%) of the participants recognised that they can sometimes forget to perform HH behaviour. For example, one of the nurses interviewed stated that “*you might get so busy and engrossed in your work that you might forget*” (P15, Nurse).

### Opportunity dimension

#### Social influences

Nurses were more likely than doctors to describe staff expressly encouraging and supporting one another in their HH compliance (*n* = 7 nurses (58.3%), *n* = 2 doctors (14.3%)). To illustrate, *“it’s just a constant reminder, the CNMs* [Clinical Nurse Managers] *would be constantly prompting you”* (P12, Nurse). Two nurses (16.7%) also mentioned intergroup conflicts, whereby a doctor or more senior member of staff reacted badly to their behaviour being corrected by a nurse. For example, *“I as a nurse would say to a doctor or a senior consultant, ‘please put on that’, they don’t like being told”* (P25, Nurse).

Doctors more frequently referred to observed social norms as an influence on their compliance as compared to nurses (*n* = 3 doctors (21.4%), *n* = 1 nurse (8.3%). An example quote of this type is, *“it’s from the top down. All the consultants wash their hands, the nurses all wash their hands and everybody else washes their hands … so there’s a culture for it.”* (P2, Doctor).

#### Environmental context and resource

Nurses in particular reported receiving direct reminders and encouragement from colleagues to engage in HH. By way of illustration, *“you remind each other. When you’re at a bedside and you go, “Will you wash your hands there before you come in?' … you’d forget sometimes, you know?”* (P15, Nurse).

Twelve (46.2%) participants described a climate in which members of staff are aware of and observe one another’s HH behaviour; participants said that this motivates compliance. One of the nurses noted that *“every single nurse will give out to you if you don’t …*. *They’ll tell you … that you need to* (P13, Nurse). Similarly, one of the doctors stated that *“there’s nurses constantly hassling you to do it* [hand hygiene]*”* (P5, Doctor).

Nurses also often described situations where material resources were lacking (*n* = 6 nurses, 50%). For example, “*in a seven bedded ward or a twelve bedded ward, you wouldn’t have sinks at every bedside.”* (P12, Nurse). Although not mentioned by doctors, nurses also described staffing levels as an occasional impediment to compliance (*n* = 8 nurses, 66.7%). For example, *“short staffed on the unit ... when people go on lunch, then there’s less people around and then things can just crack off and that can delay you.”* (P18, Nurse).

### Motivation dimension

*Social/professional role and identity*. Protecting patients through HH was recognised as part of the job. For example, “*this* [HH] *is an important part of managing your introduction to patients. It’s part of being a good doctor*” (P2, Doctor). However, it was also pointed out that nurses were less likely to miss HH opportunities as compared to doctors. To illustrate, “*I’ve noticed the nurses are quite good. Doctors, you have to prompt them a few times*” (P13, Nurse).

#### Optimism and intentions

There were no comments related to these two domains of the TDF in any of the interviews.

#### Goals

A small number of participants (*n* = 4; 15.4%) commented that the HH goal of 90% was unachievable. One nurse said, “*I do believe that if you were to do it exactly, it would be impossible, you know?*” (P18, Nurse), while a registrar described the task of perfect compliance as *“absolutely impossible … not achievable … not practical”* and said that *“if you were to follow every movement of hand hygiene you’d get no work done … you’d end up with some kind of contact dermatitis from … washing your hands forty times a day”* (P5, Doctor).

#### Beliefs about consequences

A majority of participants (*n* = 17; 65.4%) expressed the belief that poor HH contributes to infection and poor patient outcomes: “*The lack of action could harm somebody*” (P9, Doctor). However, there was also a recognition by eight (23.1%) of the participants that HH measures “*can be very hard on your hands. You’d feel like … your skin would be breaking and everything*” (P12, Nurse).

#### Reinforcement

Nine (34.6%) of the participants commented that healthcare providers are motivated to perform HH by the demonstrated or evidenced protection it offers to themselves, as well as their patients. “*As someone who works in ICU, I don’t want to get colonised with resistant bacteria either*” (P27, Doctor). Five (19.2%) of the interviewees pointed out that positive feedback from audits and infection transmission data encourages continued compliance with HH: “*If you’re looking up at the board ... and you think we’ve been free of MRSA* [Methicillin-resistant *Staphylococcus aureus*] *as acquired on the units for … that’s brilliant that you see that we’re doing something right in that sense*.” P18, Nurse).

#### Emotion

Six (23.1%) participants referred to ethical principles that encourage them to engage in hand hygiene. Commitment to caring for the patient (*“in your head you’re thinking, you know, first do no harm”* (P13, Nurse)) and reciprocity or the ‘golden rule’ (“*if I would apply that rule to myself, then I definitely should apply it to others*” (P27, Doctor)) both support good hand hygiene practices.

## Discussion

Substantial resources are invested in HH improvement efforts in ICUs [4], with considerable efforts focused upon observations of HH compliance. However, it is important that these limited resources are used effectively [21]. Although direct observation is considered the ‘gold standard’ method of measuring HH compliance [22], it only supports a very limited understanding of the reasons why a healthcare professional does, or does not, engage in appropriate HH behaviours. Therefore, there is a need to consider other complementary methods to develop an understanding of the determinants of current and desired behaviours that support HH compliance, and thereby use this understanding to develop theoretically-based and tailored interventions. The purpose of the study reported in this paper was to apply a commonly used behavioural change theory to gain an understanding of the barriers and enablers HH behaviours, and to use this information to consider the types of interventions that may be effective in improving the HH compliance of ICU staff. The discussion will consider the findings within the capabilities, opportunities, and motivation dimensions of the COM-B model and their associated TDF domains.

### Capabilities dimension

It was clear from the ICU staff interviews that the participants were confident in their capabilities to carry out appropriate HH behaviours. The vast majority of participants reported having the necessary knowledge, skills, and capabilities to perform appropriate HH behaviours. The fact that healthcare workers believe that they have the requisite knowledge and skills to carry out HH has also been found in other studies [23]. However, observations of 335 moments of HH in the two ICUs at which the interviewees worked found an overall compliance of 64% [24]. This lack of concordance between self-reported and observed HH compliance has also been reported elsewhere [25]. Therefore, although training may be required, interventions designed to increase HH knowledge or skills may not impact HH compliance, as ICU staff believe they have the requisite knowledge and skill to carry out HH when appropriate.

This perceived overestimation of HH compliance is an important barrier to engagement with training and educational interventions, and it impacts all of the TDF domains within the capability dimension of the COM-B model. It is suggested that this overestimation may partially be attributed to the fact that ICU staff generally only receive information on HH compliance at a unit level, and do not receive feedback on their own individual HH compliance. It is suggested that there is a need for targeted HH training in which ICU staff receive direct and individualised feedback on actual performance at the bedside (addresses the TDF domains of knowledge and beliefs about capabilities; see Table 1), and are provided guidance on how to address deficiencies in HH compliance at the time at which the HH behaviour was performed (addresses the TDF domains of knowledge, skills, behavioural regulation, memory, attention, and decision processes; see Table 1). Sustained individualised feedback is supported by a number of empirical studies, indicating that it improves adherence to guidelines and that it is welcomed by healthcare workers [17, 26, 27].

The influence of professional group is also important. While the doctors in our study almost universally reported confidence in their HH performance, recent research and review work indicates that physicians are often excluded from studies of HH compliance and they tend to underperform in HH compliance compared to their colleagues in nursing and allied health professions [27–30]. Professional groups respond differently to interventions [27], and so targeted profession-specific training, taking account of their particular perspectives and challenges, is an important avenue for future research to explore.

### Opportunity dimension

Social influence was regarded as being important in encouraging HH compliance by the interviewees, particularly by nurses. The sense of being watched or monitored, and explicit encouragement and reminders from peers were all identified as reinforcing or motivating factors in promoting HH compliance. In a study of the attitudes to HH of Canadian doctors using the TDF, the social environment and role models were also found to be important [3]. However, although persuasion (use of communication to produce positive or negative feelings that may promote engagement in a behaviour) is a commonly used HH compliance intervention in the ICU, a recent systematic review found no examples of the use of modelling (highlighting examples of desired behaviour in order to encourage others to emulate this behaviours) to improve HH compliance [4]. Encouraging leaders to act as ‘champions’ for HH may yield significant benefits [31]. Modelling of appropriate HH behaviours by senior leaders may be particularly important among doctors given the hierarchy and the apprenticeship model of training. A modelling-focused intervention would address both the social influences and environmental context and resources TDF domains.

The results presented here indicate some degree of mutual support between members of staff for hand hygiene; staff are reminded and encouraged to perform hand hygiene, both within and across professional groups. Research suggests that this ‘speaking up’ culture, led in our sample by women, is worth encouraging; although staff often fear speaking up due to concerns about reprisal, the perceived safety risk, and the appropriateness and perceived efficacy of speaking up [32, 33], initial efforts to speak up frequently can help hand hygiene to become part of the social norm on a ward, leading to less need to speak up over time [34].

Nurses in particular also commented on the impact of low staffing levels on HH compliance. HH compliance is a time-consuming activity that does have the potential to negatively impact patient safety if there is a shortage of staff. In an evaluation of how long nursing staff must spend in order to be 100% compliant with HH recommendations, it was found that 12 nurses working in an eight bed ICU would devote a total of 4 hours to HH if using alcohol hand gel, or 16 hours if using soap and water across an eight-hour shift [35]. Therefore, levels of staffing are a factor that must be considered if HH compliance is to be improved.

### Motivation dimension

The interviewees were motivated to carry out HH behaviours, and it was recognised that HH is an important part of their job and crucial in preventing infection. The desire to perform HH to protect themselves was mentioned by a third of the interviewees in our study. The propensity towards prioritising the protection of oneself from infection has also been found in other studies of HH compliance [28, 36, 37], including observations carried out in the two ICUs from which the interviewees in our study worked [24]. In the observations carried out in the ICUs, the staff were 55% compliant before an aseptic technique, as compared to 89% compliant after patient contact [24]. Therefore, interventions focused on those HH moments that protect the patient are merited. It is suggested that the direct observation and feedback intervention recommended above under the capabilities domain could also be an effective intervention for bringing about a focus on the HH moments that protect the patient and address the intentions, goals, and reinforcement domains of the TDF.

The *Optimism* and *Intention* domains of the TDF were not used to classify and of the participants’ comment. That all domains of the TDF were not used to describe the interview data was expected. Squires et al. [3] conducted TDF domain interviews with doctors about hand hygiene compliance and found that four TDF domains were not relevant to physician hand hygiene compliance (*Optimism*, *Intention*, *Reinforcement*, and *Emotion*). The TDF is a broad overarching approach to understanding many behaviours in a range of settings; it is unsurprising, therefore, that not all domains were relevant in this study’s specific setting.

### Limitations

The study had a number of limitations. First, only doctors and nurses were recruited using a convenience sampling approach. HH is also crucial for other members of staff, including healthcare assistants, therapists, porters, and catering staff, and it important to capture their insights also to ensure the efficacy of any intervention that follows. Second, as is common with all qualitative research, the generalisability of the findings may be questioned. Nevertheless, there is some support for the generalisability of the findings as they are in broad agreement with other studies of HH in hospital settings [2, 3, 38]. Third, the respondents may have felt some pressure to provide socially desirable responses to some of the questions. We hope that this effect was limited by the fact that the interviews were conducted by researchers from outside the hospital and the responses were anonymised. Finally, this paper only reports one method of exploring attitudes to HH compliance. This is certainly a limitation in terms of obtaining a broad understanding of HH behaviour, particularly when considering the discordance with the HH observation data. This is the very reason we recognise the importance of taking a multi-methods approach to understanding HH behaviour. In fact, these interviews were conducted as part of a larger project exploring HH compliance in Irish ICUs in which other methods of assessing HH behaviour were also utilised [4, 24, 29, 39].

## Conclusions

Best practice for improving hand hygiene in ICUs remains unestablished [4], and compliance is less than optimal [29]. Although direct observation is considered the ‘gold standard’ method of measuring HH compliance [22], it provides little understanding as to why a particular behaviour did or did not occur. This study has demonstrated that short interviews with ICU staff, founded on appropriate behavioural change theory, can provide a depth of understanding into HH behaviour that can complement, and inform, data collected from other methods (e.g. direct observation). This understanding can then be applied to the design of theoretically valid interventions that are appropriately tailored to the needs of a specific unit, and which will have an increased likelihood of success. The work presented here offers a novel, user-friendly method for infection control and quality improvement teams to explore, diagnose and understand the factors influencing HH compliance in their own units and hospitals, thereby offering an opportunity to address their specific challenges in a targeted way. This study forms part of a larger project, one aim of which is to develop a ‘toolkit’ of potential interventions that may be applied by local teams, specified according to the findings of an investigation like the one presented here. Although a ‘one size fits all’ approach is desirable from the perspective of convenience, there is a risk that, in fact, ‘one size fits nobody’, and improvement efforts that fail to acknowledge local circumstances and challenges constitute wasted opportunities to bring about meaningful long-term change.

## Supplementary information

**Additional file 1.** Semi-structured interview schedule. Description of data: Contains the schedule of questions used in the semi-structured interviews.

## Data Availability

The datasets used and/or analysed in the current study are available from the corresponding author on reasonable request.

## Abbreviations

CNM: Clinical Nurse Manager
COM-B model: Capability Opportunity Motivation and Behaviour model
HH: Hand hygiene
ICU: Intensive Care Unit
SRQR: Standards for Reporting Qualitative Research
TDF: Theoretical Domains Framework
