# Supplementary material for: Understanding hand hygiene behaviour in the intensive care unit to inform interventions: an interview study
Source: BMC Health Serv Res. 2020 Apr 25;20:353. doi: 10.1186/s12913-020-05215-4 (PMC7183607; doi:10.1186/s12913-020-05215-4)
Supplement: Supplementary file 1 — Additional file 1. Semi-structured interview schedule. Description of data: Contains the schedule of questions used in the semi-structured interviews. [file 12913_2020_5215_MOESM1_ESM.docx]

**Additional file 1: Semi-structured interview schedule**

| **COM-B Domain** | **Interview Question** |
| --- | --- |
| **Capability** | What training have you received in appropriate hand hygiene practices? |
|  | Are you confident in your knowledge of hand hygiene protocols or do you think further training or supports are needed? |
|  | What prompts are there to remind staff when and how to engage in hand hygiene practices in the ICU ward? |
| **Opportunity** | How is there a focus on encouraging adherence to hand hygiene protocol in this unit? |
|  | Do you have enough time to adhere to hand hygiene protocol for each patient or is that difficult? |
|  | What materials are necessary (e.g., sanitisation gel) for hand hygiene and are these always available to you on the ward? |
| **Motivations** | What factors hinder you from adhering to hand hygiene guidelines? |
|  | What factors encourage you to adhere to hand hygiene guidelines? |
|  | Do you think adherence to hand hygiene protocol is important for patient safety and why? |
